# Health care seeking for maternal and newborn illnesses in low- and middle-income countries: a systematic review of observational and qualitative studies

**DOI:** 10.12688/f1000research.17828.1

**Published:** 2019-02-19

**Authors:** Zohra S. Lassi, Philippa Middleton, Zulfiqar A. Bhutta, Caroline Crowther

**Affiliations:** 1 Robinson Research Institute, The University of Adelaide, Adelaide, South Australia, Australia; 2 Healthy Mothers, Babies and Children, South Australian Health and Medical Research Institute, Adelaide, Australia; 3 Centre for Global Child Health, The Hospital for Sick Children, Toronto, Canada; 4 Center of Excellence for Women and Child Health, Aga Khan University, Karachi, Pakistan; 5 Liggins Institute, The University of Auckland, Auckland, New Zealand

**Keywords:** Health care seeking, maternal health, neonatal health, developing countries, low- and middle-income countries

## Abstract

**Background:** In low- and middle-income countries, a large number of maternal and newborn deaths occur due to delays in health care seeking. These delays occur at three levels i.e. delay in making decision to seek care, delay in access to care, and delay in receiving care. Factors that cause delays are therefore need to be understand to prevent and avoid these delays to improve health and survival of mothers and babies.

**Methods:** A systematic review of observational and qualitative studies to identify factors and barriers associated with delays in health care seeking.

**Results:** A total of 159 observational and qualitative studies met the inclusion criteria. The review of observational and qualitative studies identified social, cultural and health services factors that contribute to delays in health care seeking, and influence decisions to seek care. Timely recognition of danger signs, availability of finances to arrange for transport and affordability of health care cost, and accessibility to a health facility were some of these factors.

**Conclusions:** Effective dealing of factors that contribute to delays in health care seeking would lead to significant improvements in mortality, morbidity and care seeking outcomes, particularly in countries that share a major brunt of maternal and newborn morbidity and mortality.

**Registration:** PROSPERO CRD42012003236.

## Introduction

The majority of low- and middle-income countries (LMICs) have been unable to achieve the targets set for Millennium Development Goals (MDG) 4 and 5 ^1, 2^. Even with improvements in maternal and child mortality rates over past decades, 303,000 mothers and 5.9 million children under the age of 5 years died in 2015 ^3, 4^, with 99% of these deaths occurring in LMICs. LMICs lack financial and human resources and basic utilities including clean water, sanitation and education are not always readily available. Families in LMICs often unable to access and afford health care when required, and therefore, care seeking from non-skilled birth attendants is preferred when women give birth.

Rates of birthing at home are higher in LMICs, and usually skilled birth attendants (SBA) are not present ^5^. In Sub-Saharan Africa, 50% of births occur at home with no skilled birth attendant; in South Asia, mothers and their families are the primary care givers of a third of all home births. In these regions, the inequalities are even higher among poorer people, particularly those living in very remote geographical areas ^6^. While interventions to reduce poverty may require more time, training and deploying skilled birth attendants and upgrading emergency obstetric care are urgently needed ^7^. Evidence suggests an association of skilled birth attendance with reduced neonatal mortality—77% of neonatal mortalities occur where coverage of skilled birth attendance is 50% or lower ^8^.

While a systematic review that assessed the determinants of skilled attendance or health facility use for delivery in LMICs has been performed ^9^, there was no attempt to identify the barriers and facilitators of health care seeking for maternal and newborn illnesses in LMICs. Another systematic review on effectiveness trials that has also identified strategies that can improve maternal and newborn health care seeking ^10^; however, a review of narrative and qualitative studies is required to identify barriers and enablers of health care seeking in LMICs. We aimed to systematically review observational and qualitative studies to identify factors associated with delays that lead to serious maternal and neonatal morbidity and mortality ^11^. These delays occur at three levels: 1) identification and decision making to seek care; 2) arranging means to reach a health facility; 3) receiving adequate care at the health facility.

## Methods

All observational and qualitative studies from LMICs that assessed health care seeking behaviour or pattern for maternal and newborn health care and illnesses were included. We define health care seeking as ‘sequence of remedial actions that individuals undertake to rectify perceived ill-health’. The primary aim was to identify the barriers and enablers of maternal and newborn health care seeking and related pathways in LMICs. The protocol for this systematic review and meta-analysis has been registered with PROSPERO 2012: CRD42012003236.

### Search strategy

The search engines PubMed, Medline, EMBASE, the Cochrane Library, and Google Scholar were initially searched up to Sep 16, 2016 and then searches were revised on September 27, 2017, but we found that data was saturated, and no new themes were emerged. Search terms were a combination of [(‘care seeking’ OR ‘care-seeking’ OR ‘health care’ OR ‘health care seeking’ OR ‘community based intervention*’ OR ‘community-based intervention*’) AND (mother* OR maternal OR women OR newborn* OR neonat*)] used as medical subject headings and keyword terms in the title/abstract. No language restrictions were applied. Grey literature and reference lists of included studies were also searched to identify studies. We considered studies from LMICs that assessed the factors associated with health care seeking for maternal and newborn illnesses in observational or qualitative studies. We did not consider studies on health care seeking for specific maternal and newborn illnesses such as jaundice etc. or for preterm babies. We considered recommendations for systematic reviewing of qualitative studies ^12^. We used the PRISMA checklist PRISMA statement in reporting systematic reviews from the observational studies ^13^. The 22-items STROBE checklist was used to assess the methodological quality of the cross-sectional studies ^14^. Studies that fulfilled the methodological criteria of more than 18 points were classified as high quality, between 12–18 as moderate quality and below 12 were classified as low quality.

ZSL and PM independently reviewed the retrieved articles in two stages. First, relevance was assessed from the title and abstract and if relevance was still unclear, the full text was read. Any disagreement was referred to a third reviewer (CC or ZAB). Factors responsible for health care seeking patterns for maternal and newborn health from observational studies and qualitative studies were separately analysed. Study design, country of study, setting, participants, and results were recorded for each study. We performed a narrative synthesis of the findings from the included studies, as included studies were observational and qualitative in nature.

## Results

**Figure 1.**
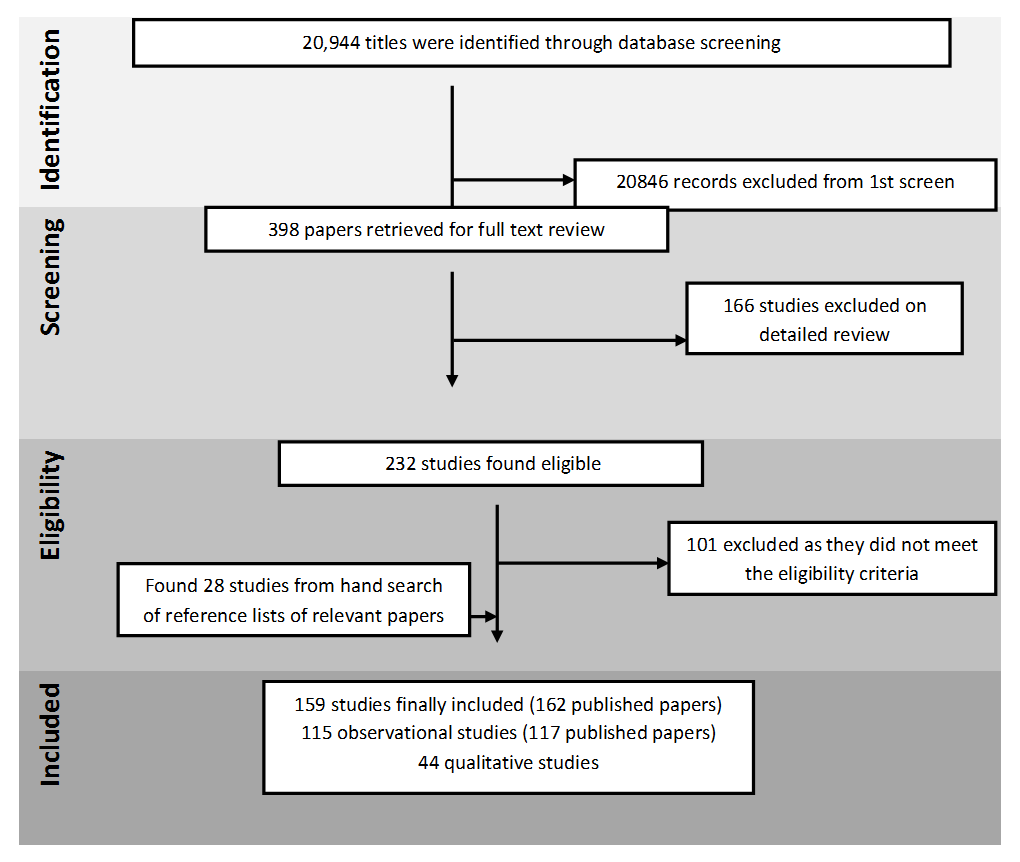
Search flow diagram.

Our search strategy identified 20,944 articles, of which 232 met the inclusion criteria ( Figure 1). We found and analysed 159 original studies (162 published papers), of which 115 were observational studies and 44 were qualitative studies (characteristics of included studies are included as extended data ^15^. Observational studies were moderate to high in quality upon quality assessment.

## Qualitative findings for delays in care and pathways of care seeking

Health-care-seeking patterns are complex phenomena, often confounded by several interlinked factors such as education of mothers, socio-economic status and age. More than half of the included observational studies reported that poor maternal health care utilization and giving birth at home is associated with lack of antenatal care, age, parity, education and employment status of women ^13, 16– 74^. On the other hand, seeking care for newborn illnesses depends on the severity of illness ^75– 80^ and gender of the baby, with preference being given to male children ^48^. Studies have reported that adequate health care seeking from skilled health care providers leads to fewer deaths and morbidities ^81– 83^. Women who had good marital relationships with husbands were more likely to report receiving antenatal care and institutional birth ^55, 56, 84– 90^. Similarly, women who had good relationships with their mothers-in-law reported being able to attend or receive antenatal care, with the degree of bonding and communication of women with their mothers-in-law reported to be an important influencing factor ^91^. We identified several qualitative themes in the section below that describe the reasons for delays in health care seeking and associated pathways. 

### Identifying the illness and first preferred level of care

Primary caregivers in all included studies were usually mothers; however, mothers-in-law, grandmothers, fathers, neighbours, traditional healers and opinion leaders in the community were among the many people involved as caregivers for mothers and babies. Across the studies, it was observed that mothers/families do seek care for neonatal illness ^80^; however, complications during pregnancy are not considered as an illness and many signs are considered as normal, even when painful and constant ^92– 102^. In certain studies, bleeding was not considered to be a complication ^103^, and in such situations decisions to seek health care were often delayed. Women who expressed pain verbally were considered as disobedient and therefore maintaining silence was considered appropriate ^104, 105^. Missing antenatal care visits were reported to be due to heavy and unavoidable workloads at home ^91^, and a few studies reported that mothers-in-law privileged household chores over women’s health ^91, 99^. Some families perceived that some common neonatal symptoms should or cannot be treated at health facilities and therefore traditional care should be sought ^106^.

In India, women during pregnancy are usually advised to be cautious while eating “hot” or “cold” food, and to eat less otherwise the baby can grow too large and therefore lead to a difficult birth ^92^. A qualitative study from Pakistan (Baluchistan) ^107^ described that the *dai* (traditional birth attendant (TBA)) usually places mustard oil on her fingers and massages the vaginal walls to ease the birth, and inserts vaginal and anal pessaries after birth to help shrink the uterus and to provide support for the uterus and backbone. They also prefer women to eat *Goandh* (edible gum) combined with turmeric powder, and dried dates in milk to induce heavy vaginal bleeding so that all unclean blood is drained from the body, thus predisposing to postpartum haemorrhage. In situations when the placenta does not expel normally, the *dai* enters her bare hands in the uterus or puts hair into the mother’s mouth to induce vomiting ^103^. Eating vegetables rather than meat during pregnancy is preferred as it is considered to increase the production of breastmilk and freshens its taste ^108^. During infant illnesses, mothers prefer to give ‘ *rabadi*’ ** (prepared by cooking millet flour and yogurt), ‘ *khichchadi*’ (a semi-liquid rice and pulses mixture) and *‘mateera*’ (watermelon curry) to their febrile children in conjunction with breastmilk ^109^.

While illnesses, particularly in women who are not pregnant, are considered unimportant, evil spirits and fate (Allah’s will) are reported to be the cause of these illnesses ^103, 110^. Faith healing is important in many cultures. A study from Ghana ^111^ named three major religions that practised faith healing and each religion has a specific healer. On the other hand, most of the communities in Asia and Africa believe that certain precautions during pregnancy or immediately after birth will ward off the evil eye (a gaze or stare superstitiously believed to cause harm) and will prevent the infant from getting sick ^92, 112^. This includes isolating women and their baby in a room for a certain period of time after childbirth and lighting a fire at the entrance where they are confined ^92^. In order to prevent them from evil eyes, people reported keeping the pregnancy secret from people outside of close relations ^96^. 

Mothers may consult family and friends when the danger signs are not clear or unusually severe ^107, 113^. However, in severe illness, decision-making power can be switched to more experienced members of the extended family, which can cause significant delays in decision-making. Many of the studies reported that in scenarios when women had money, they hurried to pursue treatment options from a health care facility despite several familial pressures. A study from Tanzania reported ^95^ that having an option of home birth was found to be a hurdle in emphasizing the importance of skilled birth care ^100, 104, 114– 116^. Trust for someone from the same community, sharing the same values and speaking the same language, was another factor that encouraged women to give birth at home and with a TBA ^38, 117^. However, it was apparent from the studies that if women continued to suffer, then they do seek care from western-trained care providers ^107^.

### Barriers on deciding to seek care for illnesses and choice of care

Decision-making emerged as a complex issue. Decision-making power is less likely to be with the woman and mostly rests with their partners and mothers-in-law. Women who had no income source were usually those who had no rights for decision making ^118^. Several studies reported that the major barrier for institutional care was gaining permission from husbands ^92, 93, 104, 119– 124^. Women are considered inferior to men and their disobedience often results in physical and emotional violence ^123^. If husbands are absent, women face difficulties in receiving permission from her husband’s parents or other elders for seeking care and this results in even greater delays. Husbands and elders often have control over finances and women are mostly dependant on them ^38, 93, 99, 108– 110, 117, 121, 122, 125– 131^. Deciding to seek care can incur transportation costs, user fees, cost of medicines, and possibly ensuing costs of misdiagnosis and treatment failures ^93^. Considering all these barriers, women often postpone seeking help, with the hope that the problem will subside on its own.

When a family is willing to seek care and arranges the money required, other challenges such as physical transference of mothers and newborns to health facilities becomes a problem. The situation is even worse if complications arise at night, when risk of being attacked by criminals’ increases or when transport providers raise their taxi or car-hire charges ^96, 118, 121, 122, 125, 127, 130– 143^. Studies on people living in very remote areas reported factors such as distance to health facility and related transportation issues, lack of financial resources, encountering swollen rivers on the way, fear of encountering wild animals, shame about too many pregnancies or being of advanced age and pregnant as some of the critical reasons for not seeking care. Studies also reported other factors responsible for not seeking health care such as non-availability of staff at facility, rude behaviour of health care staff, and poor quality of care ^96^. Fear of operative procedures was reported as a factor hindering care-seeking ^144^. These were usually based on previous experience and contact with health care staff and the health care service received ^93, 122, 125, 145, 146^.

Cost is another important barrier to seeking care from trained health professional. However a study from rural Mexico reported that cost of care from TBAs is sometimes higher than facility birth but women prefer them because they can give birth at home ^147^. Many women also preferred giving birth at home because they preferred a squatting position for giving birth that was also endorsed by TBAs ^147^. Relatives being not allowed at facilities during the childbirth was another factor expressed for giving birth at home ^147^.

Women’s previous encounters with health care staff and facilities were reported as a key factor for decision making ^148^. Further, many of the danger signs are not considered as pregnancy-related complications ^38, 80, 109, 113, 117, 118, 149^, and thus families seek help from traditional healers, community health workers or drug sellers. Households often regard accessible and less expensive care such as herbal and home remedies or locally available drugs more highly ^150^. Workers from these types of care were often praised as they give time to patients and consider their social and cultural aspects as well.

### Receiving adequate care when facility is reached

Women and families usually opt for medically qualified birth attendants where women are perceived to have possible birth complications. Where TBAs detected a complication at home, women were provided with referrals. Women also preferred SBA when they wanted to have a tubal ligation performed ^147^. Studies reported that perceived fear of being torn in hospital, where Caesarean section was required ^151^ discourages women to seek institutional care for childbirth ^104^. Lack of privacy at care facilities and being examined in the open are other factors for not seeking care at clinics ^152^.

Pregnant women or mothers with ill newborns usually experienced long waiting times when seeking hospital care ^93, 104, 153, 154^. Most of the included studies cited that health professionals have poor attitudes towards poor or pregnant women, which are stigmatizing ^118, 131, 144, 145, 155^. Studies pointed out that health care staff examine women in hurry and at many occasions did not clarify their concerns. Staff may stigmatize women, criticising them for their age and number of pregnancies and judge them on their practices for family planning ^152^. Staff behaviour is therefore a major barrier for accessing care ^151^.

Sometimes, women are referred to another facility due to lack of trained staff and functional equipment and supplies that lead to further delays. Women may be asked to pay for fuel for the ambulance to take them to the other facility. They then may be required to pay for medicines and other supplies, and when stocks of these run out, there are further delays in receiving care ^118^.

## Discussion

**Figure 2.**
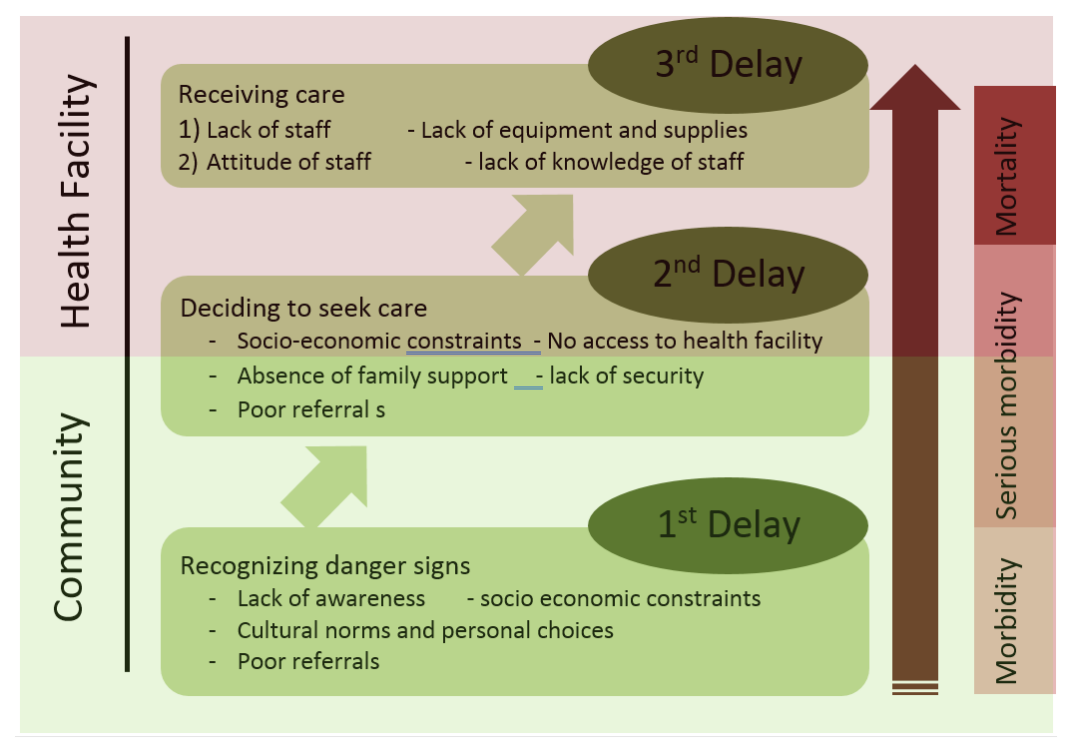
Factors associated with delays in seeking care for maternal and newborn illnesses.

**Figure 3.**
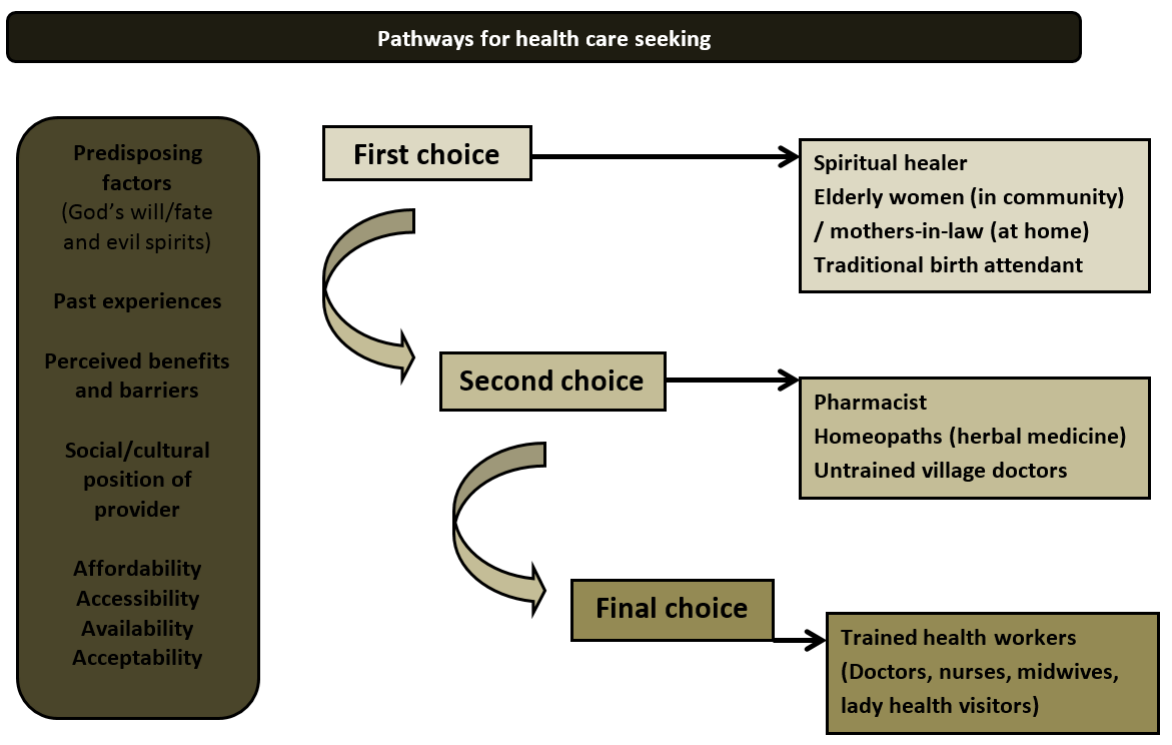
Pathways for health care seeking.

It is often suggested that overwhelming maternal and neonatal mortalities and morbidities are closely linked with a number of interrelated delays that prevent a pregnant women or neonate from accessing the health care needed ^11^. Each delay is closely related to services, logistics, facilities and conditions. Our review identified factors associated with delays ( Figure 2) and the pathways for health care seeking in cases of illnesses ( Figure 3). Although the pathways of seeking care were not similar across all the studies, choices usually followed the same pattern if not the same levels. Depending on predisposing factors (be it God’s will, past experiences, user affordability, accessibility, availability or acceptability), the first choices for seeking care were for spiritual healers and immediate elder members of the family and community such as mothers-in-law and TBAs, who not only hold a respected position in the community but are generally considered as experienced and knowledgeable people. If not gaining any benefits from the care received from the first level, women then consult pharmacists, homeopaths and quack healers or untrained village doctors. However, the last choice (can be second or third) is usually the trained doctors, nurses or lady health visitors in health facilities.

Ineffective or inequitable decision making was reported as the biggest hurdle for seeking care during illnesses. Several cultural, economic, and health system related factors confound this further. Prompt identification of danger signs, autonomy of decision making, availability of finances, accessibility to health facility, and perceived quality of care play a major role in institutional health care seeking. Distance and cost were highlighted as the two main reasons for causing delays in decision making. Inadequately equipped facilities further delays care ^156^. Improvement in medical care seeking can be achieved if behaviour change communication interventions are contextualized and meet specific needs of the community. Similar findings have been reported by an earlier review on determinants of skilled birth care and institutional births ^9^.

This review highlights the reasons for delays and the ramifications of these delays on morbidity and mortality outcomes. Delays at each level serve as barriers, and strategies to overcome these may help and empower the communities to select and make early decisions. Cultural norms, societal values along with limited financial resources were underscored as major hindering factors for care seeking. It is therefore important that health system reforms related to maternal and newborn health should consider societal and cultural barriers and practices to improve their health care seeking. A major obstacle is women’s self-sufficiency and lack of empowerment to make decisions about their health. A change brought about in the attitude of the family members with emphasis on the need for women’s autonomy in making these crucial health decisions will have an immediate positive impact. Women should have the right to choose where they give birth, although it is important to help the woman comprehend the risks associated with these options. This could be achieved by proper mobilization of the entire family. At the same time, health systems should train health workers to provide and manage emergencies. A specific implementation strategy could be the provision of birthing kits to the TBAs which will ensure access to this facility to those residing in remote areas. This will reduce mortality arising from delay in the provision of emergency medical aid during childbirth. In addition, government should subsidize health care costs and should introduce schemes such as conditional cash transfers particularly for places where access to health care facility is an issue. These remedies have also been found to be cost-effective ^157^.

While we were able to extract the important factors associated with maternal and newborn health care seeking, the review also faced some methodological challenges. First, the findings from the observational studies need to be interpreted with caution as included studies employed different inclusion criteria. Second, the studies used different statistical modelling to control for confounders and clustering therefore made it hard to compare the results. Third, the findings, particularly from the qualitative studies, were from different geographical settings and the barriers faced in one community may not exist or differ in another community. Therefore, strategies to improve health care seeking need to be context- and community-specific. Earlier review of experimental studies suggested that simple strategies such as community mobilization and home visitation via community health workers may improve health care seeking and perinatal survival ^10^. Our findings from the observational and qualitative studies have identified the important barriers of health care seeking that need to be considered while developing strategies.

## Conclusion

Despite all the progress made towards improving maternal and newborn health in past few decades, many LMICs could not reach the MDGs. This review has identified several socio-economic, cultural and health services related factors that contribute to delays in health care seeking. Effective implementation of strategies after controlling for these factors of delays such as increasing women’s autonomy would lead to significant improvement in mortality, morbidity and care seeking outcomes.

## Data availability

### Underlying data

All data underlying the results are available as part of the article and no additional source data are required.

### Extended data

Open Science Framework: Health care seeking. https://doi.org/10.17605/OSF.IO/5UT6X ^15^. Supplementary Files contain information concerning characteristics of the studies included in this review.

### Reporting guidelines

Open Science Framework: PRISMA 2009 Checklist for this study. https://doi.org/10.17605/OSF.IO/5UT6X ^15^.

Data are available under the terms of the Creative Commons Zero “No rights reserved” data waiver (CC0 1.0 Public domain dedication).
