# Supplementary material for: Health care seeking for maternal and newborn illnesses in low- and middle-income countries: a systematic review of observational and qualitative studies
Source: F1000Res. 2019 Feb 19;8:200. [Version 1] doi: 10.12688/f1000research.17828.1 (PMC6480947; doi:10.12688/f1000research.17828.1)
Supplement: Peer review history

# Peer review: Reviewer response for version 1

**DOI:** 10.5256/f1000research.19492.r44643

**Published:** 2019-04-23

**Authors:** Alfonso Rosales

**Affiliations:** 1 World Vision US, Washinton, DC, USA

This systematic review of maternal and newborn care-seeking practices adds important information regarding maternal and newborn care seeking patterns and associated barriers.

 With its methodology clear and sound, results provide important findings to support future health program design in service provision for mothers and newborn.

 One important aspect not clearly addressed, in the results and discussion section, is related to the difference or lack of thereof between maternal and newborn care-seeking pathway. There seems to be published evidence on this subject, which seems to lead on the different care-seeking pathways, with consequences in future program design. Authors, may want to include a special issue on this theme published at the Journal of Health, Population and nutrition in December of 2017.

 The conclusion, seems to be rather short and not conclusive enough for relevant findings. It leaves out important common factors such as the need to include community/user context for strategy/intervention design, for example.

I have read this submission. I believe that I have an appropriate level of expertise to confirm that it is of an acceptable scientific standard.

# Peer review: Reviewer response for version 1

**DOI:** 10.5256/f1000research.19492.r45478

**Published:** 2019-03-25

**Authors:** Aastha Kant

**Affiliations:** 1 Harvard Global Research Support Centre India (HGI), Mumbai, Maharashtra, India

The present article is a systematic review of qualitative and observational research studies on care seeking for maternal and newborn illness in low and middle income countries. There has been a knowledge gap in synthesizing studies to understand barriers and facilitators in the context of the Three Delay Model and pathways of care seeking.

 The literature search has been conducted in a robust manner. The authors could expand a little more on 'narrative synthesis' of the findings in the context of qualitative and observational studies. 

 The authors have included literature search up to September 2017. A special issue of the Journal of Health, Population and Nutrition (December 2017), focuses on illness recognition and care seeking pathways of maternal and newborn illness in seven countries. The authors might like to update the search to include more recent articles. 

 The authors have synthesized the findings of the studies, combining maternal and newborn illness. They may want to present the care seeking pathways for maternal and newborn illness independently to explore the effect of the timing of the onset of illness (during pregnancy, childbirth or post childbirth for maternal illness, for instance) on care seeking behavior. This will also allow the authors to explore how the different perceptions of the causes of illness- supernatural or medical - often influence care seeking pathways.

 Overall, this is a very relevant work that can add to the body of knowledge in the context of maternal and newborn health.

I have read this submission. I believe that I have an appropriate level of expertise to confirm that it is of an acceptable scientific standard.
